# Imaging Review of Angiotensin-Converting Enzyme Inhibitor-Induced Angioedema of the Head and Neck

**DOI:** 10.7759/cureus.14021

**Published:** 2021-03-21

**Authors:** Saif Baig, Rachel Stein, Dalys Haymes, Peter Fiester, Dinesh Rao

**Affiliations:** 1 Radiology, Nassau University Medical Center, East Meadow, USA; 2 Neuroradiology, University of Florida Health, Jacksonville, USA

**Keywords:** computed tomography, head and neck, angioedema, ace-i, swelling, imaging

## Abstract

Angiotensin-converting enzyme inhibitors (ACE-i) are commonly used medications to treat hypertension and congestive heart failure. Angioedema is a well-established side effect of ACE-i and most commonly manifests as swelling of the mucosal and extra-mucosal soft tissues in the head and neck. CT with contrast is generally used to evaluate for airway compromise and to exclude other etiologies of edema. Herein we present five cases that illustrate the radiological findings specific to ACE-i-induced angioedema on enhanced CT scans.

## Introduction

Angiotensin-converting enzyme inhibitor (ACE-i)-induced angioedema is a self-limiting but potentially life-threatening side effect of ACE-i use. Angioedema is defined as the swelling of the deep layers of the skin or mucosal surfaces [1]. In the context of ACE-i-induced angioedema, edema is thought to occur from the inhibition of ACE-mediated bradykinin and substance P degradation. Bradykinin and substance P are inflammatory mediators, released by the contact activation pathway. These inflammatory mediators cause vasodilation and increase vascular permeability, resulting in plasma extravasation into the skin, subcutaneous tissues, and submucosa [2]. Normally, ACE will degrade bradykinin and substance P prior to the development of life-threatening angioedema; however, this protective mechanism is inhibited by ACE-i therapy (Figure 6).

ACE-i angioedema accounts for one-third of all angioedema-related visits to the emergency department (ED) [2] and is associated with a 0.7% incidence rate within the first month of prescription and 0.23% within one year [3]. Incidence peaks within the first month of treatment, with the risk of angioedema decreasing significantly after 9-12 weeks [3]. However, it is important to note that the risk of ACE-i angioedema persists even after many years of use [2,3].

We present a case series to illustrate the imaging manifestations specific to ACE-i angioedema of the head and neck. 

This article was previously presented as a meeting abstract at the 2020 European Congress of Radiology Summit in July 2020 (DOI: 10.26044/ecr2020/C-15054).

## Case presentation

Institutional Review Board (IRB) approval for the study was obtained from the University of Florida Health IRB committee prior to conducting retrospective chart reviews (IRB202100494). Informed consent requirements were waived. We used the mPower search engine to look for ACE-i angioedema and identified 30 patients with the descriptive characteristics of the condition in their imaging reports. Based on the chart review, five patients were deemed to be clinically diagnosed with ACE-i angioedema. No identifying information is included in the following case descriptions or figures.

Case 1

**Figure 1:**
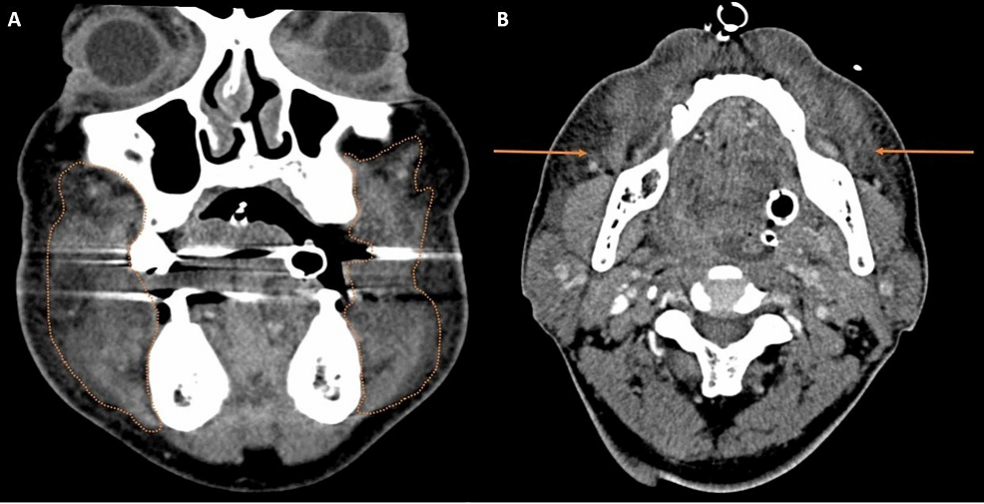
Coronal and axial enhanced CT A: Coronal enhanced CT demonstrating buccal mucosal swelling bilaterally representing angioedema (orange outline). B: Axial enhanced CT demonstrating findings of superficial and deep subcutaneous fat stranding representing subcutaneous angioedema (orange arrows). Evaluation of the tongue was limited due to beam hardening from dental amalgam and distortion from the endotracheal tube CT: computed tomography

A 62-year-old African American male with a history of hypertension on ramipril, initiated six weeks prior, presented with physical findings of swelling of the face, lips, and tongue. The patient had previously taken a medication, which had made him swell all over his body, and had been subsequently advised to avoid taking that medication. He could not remember the name of the medication that had caused these symptoms previously. At that time, he had been treated with antihistamines, steroids, and a proton pump inhibitor. On this presentation, his blood pressure was noted to be 187/90 mmHg, and WBC was elevated at 12.7. The remainder of the vital signs and lab values were normal. Over the course of two hours in the ED, the patient was noted to have a progression of the lip and oral cavity mucosal swelling and underwent oral rapid sequence intubation (RSI) with a video laryngoscope. He was found to have vocal cord edema. A CT of the neck was ordered to evaluate for structural lesions (Figure 1). The patient was treated with intravenous (IV) methylprednisolone and diphenhydramine and extubated one day later with complete resolution of symptoms.

Case 2

**Figure 2:**
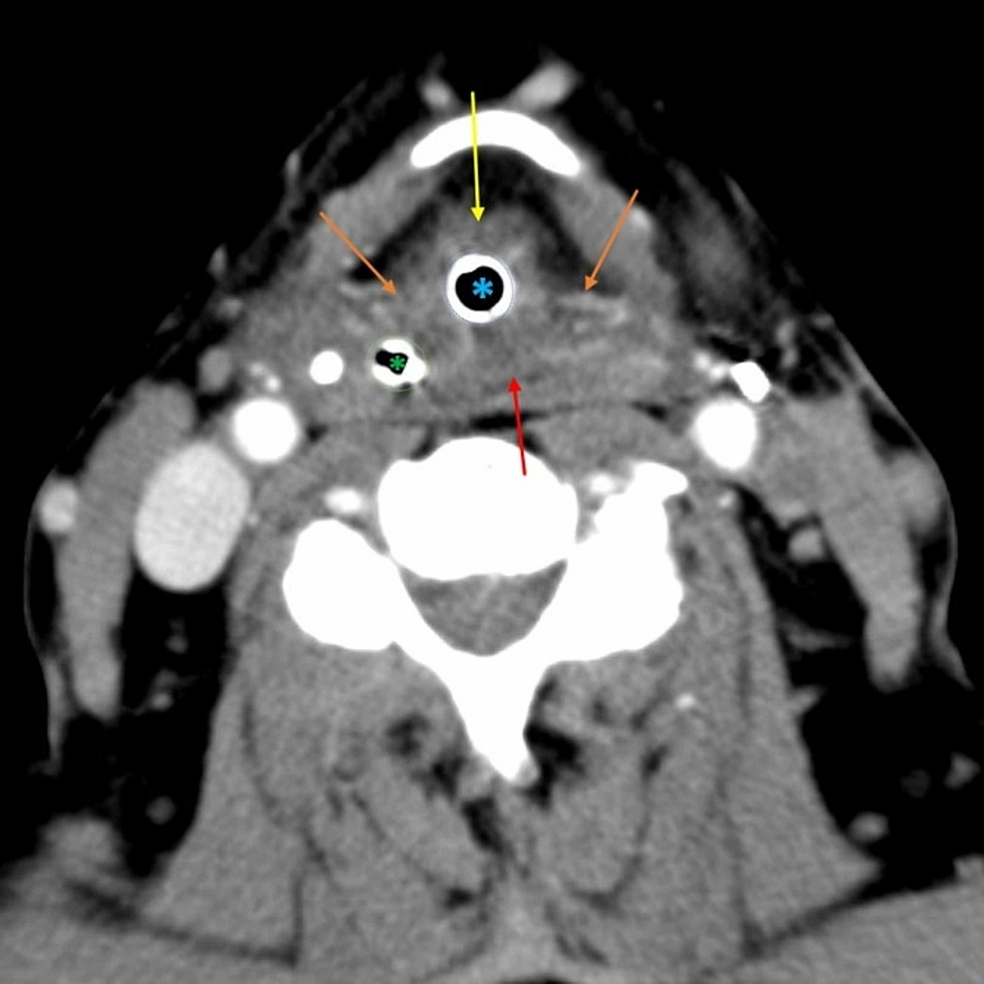
Axial enhanced CT The image shows edema of the aryepiglottic folds bilaterally (orange arrows), posterior hypopharyngeal wall (red arrow), and swelling of the epiglottis (yellow arrow). The presence of the endotracheal tube (blue asterisk) and nasogastric tube (green asterisk) distort surrounding mucosal anatomy CT: computed tomography

A 59-year-old African American male with a history of hypertension on lisinopril, initiated three days prior, presented with worsening dysphagia, sore throat, and shortness of breath for 24 hours. He also complained of a muffled voice and was noted to have tachypnea and tachycardia. He was normotensive at presentation. The patient received epinephrine upon presentation. He demonstrated a prominent uvula and underwent emergent nasal intubation for respiratory distress with a video laryngoscope, at which time his true vocal cords were noted as swollen. The epiglottis was also noted to be swollen. The patient had an elevated WBC of 13.6 but was afebrile. The remainder of the laboratory values were noncontributory. A CT of the neck with contrast was ordered (Figure 2). He was treated with IV methylprednisolone, diphenhydramine, fresh frozen plasma, ranitidine, and ceftriaxone. The patient recovered two days after the presentation and was extubated.

Case 3

**Figure 3:**
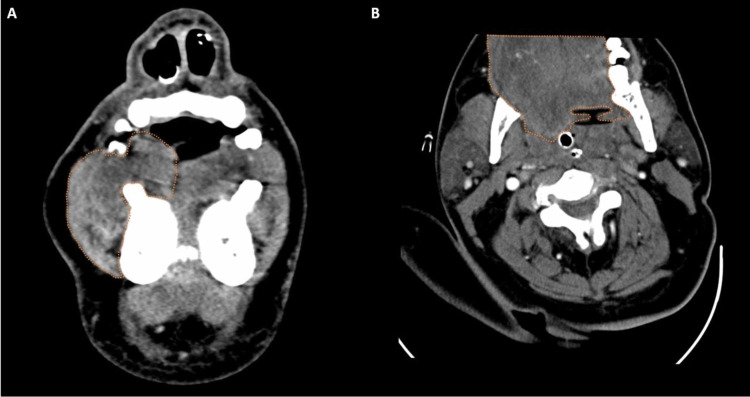
Coronal and axial enhanced CT A: Coronal enhanced CT demonstrating oral tongue and buccal mucosal swelling (orange outline). B: Axial enhanced CT demonstrates oral tongue edema (orange outline) CT: computed tomography

A 70-year-old African American male with a history of hypertension and end-stage renal disease on lisinopril for 11 years presented to the ED with physical exam findings of mild drooling as well as tongue and submandibular swelling. The patient was initially nasally intubated due to concern for airway compromise; however, he did not demonstrate symptoms of respiratory distress with an oxygen saturation of 99% on a non-rebreather mask. His blood pressure was noted to be 138/92 mmHg on presentation with mild tachycardia of 110. The WBC was elevated at 16.7. However, the patient became increasingly edematous in the ED, and therefore the nasotracheal tube was removed and he underwent subsequent endotracheal intubation. A CT of the neck was ordered to rule out infection (Figure 3). Lisinopril was discontinued and the patient was treated with IV methylprednisolone, diphenhydramine, and famotidine. He was extubated two days later.

Case 4

**Figure 4:**
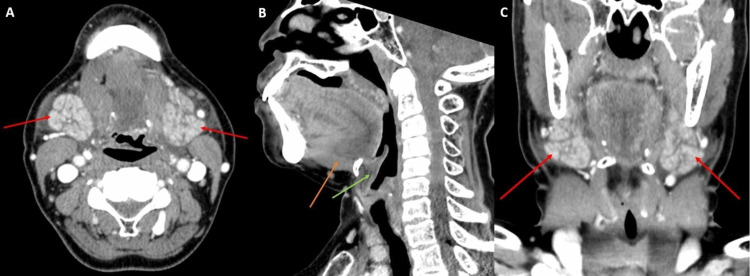
Axial, sagittal, and coronal enhanced CT A: Axial enhanced CT demonstrates submandibular gland swelling (red arrows). B: Sagittal enhanced CT demonstrates pre-epiglottic fat stranding (green arrow) and base of tongue swelling (orange arrow). C: Coronal enhanced CT demonstrates submandibular gland and surrounding submandibular space swelling (red arrows) CT: computed tomography

A 60-year-old African American female with a history of hypertension, chronic kidney disease, and coronary artery disease on lisinopril for five months presented with complaints of neck swelling and difficulty swallowing upon waking up. Initial blood pressure was noted to be 144/88 mmHg and WBC was 5.87. She was observed to have prominent bilateral submandibular glands and submandibular space swelling on palpation. A CT of the neck with contrast was performed (Figure 4). The patient was treated with IV dexamethasone, omeprazole, ketorolac, and two doses of clindamycin. Lisinopril was discontinued. Her swelling improved during her ED course and she did not require intubation. The patient was discharged home and she followed up with her primary care physician who noted complete resolution of symptoms one week later.

Case 5

**Figure 5:**
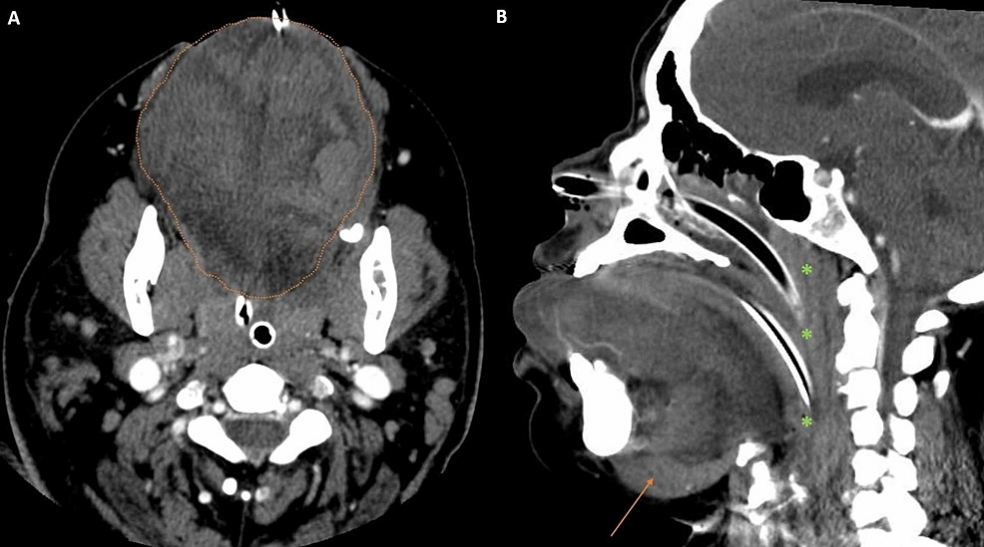
Axial and sagittal enhanced CT A: Axial enhanced CT demonstrating severe oral tongue swelling (orange outline). B: Sagittal enhanced CT showing oral tongue protruding through the oral cavity anteriorly and inferiorly displacing the mylohyoid (orange arrow). Edema of pharyngeal mucosa causing obstruction of naso- and oropharynx (green asterisks) is also seen CT: computed tomography

A 50-year-old African American female with a history of diabetes and hypertension who had been treated with lisinopril for five months recently underwent a Caldwell-Luc procedure for left maxillary fungal sinusitis. The patient was discharged after surgery on antifungal medication. The patient returned to the ED after a witnessed seizure and epistaxis. She developed rapid-onset angioedema and was noted to have severe oral tongue swelling on physical exam. She was emergently intubated for airway protection. CT of the face was obtained to assess for residual or recurrent sinus infection (Figure 5). Severe oral tongue swelling was noted. Lisinopril was discontinued and the patient received prednisone. The patient was successfully extubated the following day and the oral tongue angioedema resolved over the course of five days.

Pertinent information related to each case is summarized in Table 1.

**Table 1:** Clinical and radiographic features of ACE-i angioedema case series ACE-i: angiotensin-converting enzyme inhibitor; M: male; F: female; IV: intravenous; D/c: discontinued; SOB: shortness of breath

Case	Age (years)	Gender	Race	Duration of ACE-i use	Presenting symptoms	Intubation requirement	Pertinent imaging features	Treatment	Days intubated
1	62	M	African American	6 weeks	Swelling of face, lips, and tongue	Yes	Vocal cord edema, buccal mucosal swelling, deep subcutaneous fat stranding	D/c ACE-i, IV methylprednisolone, diphenhydramine	1
2	59	M	African American	3 days	Dysphagia, sore throat, SOB	Yes	Edema of aryepiglottic folds, posterior hypopharyngeal wall, and epiglottis	D/c ACE-i, IV methylprednisolone, diphenhydramine, fresh frozen plasma, ranitidine, and ceftriaxone	2
3	70	M	African American	11 years	Drooling; tongue and submandibular swelling	Yes	Oral tongue and buccal mucosal swelling	D/c ACE-i, IV methylprednisolone, diphenhydramine, and famotidine	2
4	60	F	African American	5 months	Neck and submandibular swelling, difficulty swallowing	No	Submandibular and base of tongue swelling, pre-epiglottic fat stranding	D/c ACE-i, IV dexamethasone, omeprazole, ketorolac, and two doses of clindamycin	0
5	50	F	African American	5 months	Seizure, epistaxis, oral tongue swelling	Yes	Severe oral tongue swelling and protrusion, edema of pharyngeal mucosa	D/c ACE-i, prednisone	1

## Discussion

Angioedema, also known as Quincke’s disease, was first described in the setting of isolated angioedema of the uvula [4] but is now commonly defined as the swelling of the deep layers of the skin or mucosal surfaces [1]. There are multiple etiologies of angioedema, with the main causes being allergic or nonallergic [5,6]. Drug-induced angioedema is classified as a type of allergic cause, with ACE-i being the most common offender, accounting for up to 40% of all cases [7]. Other etiologies include hereditary or acquired C1 esterase inhibitor deficiency, hereditary angioedema, and acquired angioedema, a rare condition associated mainly with lymphoproliferative disorders and autoimmune diseases [8]. Additional medications may also cause angioedema, including rituximab, alteplase, fluoxetine, laronidase, lepirudin, and tacrolimus [9]. In rare instances, isolated ACE-i angioedema of the small bowel mucosa can occur, causing diffuse bowel wall thickening, ascites, and small bowel obstruction [10].

Clinical history and specific medication history are crucial for reaching the correct diagnosis in the setting of ACE-i angioedema. Patients will typically present with swelling of the tongue, lips, face, and throat and maybe in respiratory distress. While the diagnosis of angioedema is primarily clinical, imaging plays an important role in evaluating for airway obstruction and excluding other causes of swelling such as pharyngitis, peritonsillar abscess, epiglottitis, ascending mediastinitis, and neoplasms [11]. CT with contrast is often the initial imaging modality given its widespread availability in the ED and rapid assessment. The diagnosis in all of our cases was made after direct laryngoscopy and CT with contrast were acquired. On imaging, it is important to keep in mind that ACE-i angioedema, as opposed to angioedema of other etiologies, most frequently affects the oral cavity mucosa, pharyngeal mucosal space, and larynx, as illustrated in our case series [12]. This can result in airway compromise, and emergent intubation may be necessary, leading to a higher risk of ICU admission for affected patients [2,3].

**Figure 6:**
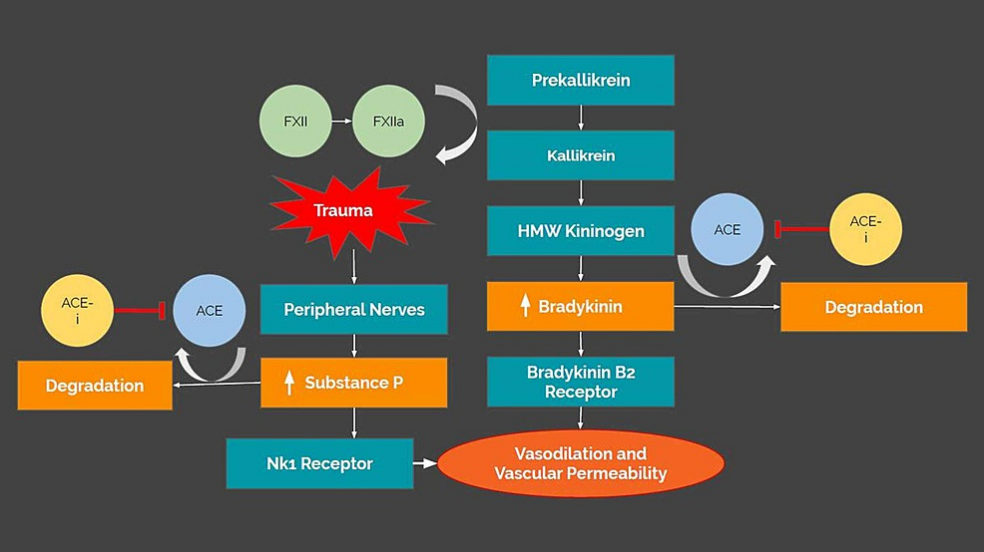
Bradykinin and substance P degradation pathways The contact activation pathway is responsible for bradykinin release and peripheral nerve activation in the setting of trauma or noxious stimuli, releasing substance P. Bradykinin and substance P mediate vasodilation and edema through NK1 and beta-2 receptor activation. ACE-i inhibits ACE-mediated degradation of bradykinin and substance P ACE: angiotensinogen-converting enzyme; ACE-i: ACE inhibitor; FXII: factor XII; FXIIa: factor XIIa

Airway compromise is defined as patients experiencing airway obstruction, hypoxia, and hypoventilation [13]. In emergent airway compromise, RSI is the accepted standard for airway rescue and protection, by utilizing fast-acting neuromuscular blockade and direct laryngoscopy in order to place an endotracheal tube [13]. Four of our five cases received RSI, highlighting the life-threatening nature of ACE-i angioedema and the necessity of evaluating early signs of airway compromise on imaging. After the airway is assessed and the patient stabilized, management involves discontinuing the ACE-i, and the angioedema typically resolves within 48-72 hours [6]. Several of our patients received steroids and antihistamines; however, these medications usually fail to rectify the underlying pathophysiology of bradykinin accumulation (Figure 6) [14]. Therefore, to date, there is no established treatment protocol for ACE-i angioedema other than airway protection and discontinuation of the ACE-i. While bradykinin receptor antagonists could be helpful in theory, they have failed to show clinical efficacy so far [15].

## Conclusions

ACE-i-induced angioedema most commonly manifests in the head and neck. Most cases of ACE-i angioedema occur within four weeks of drug-use onset. Long-term use also carries a cumulative risk. Contrast CT of the head and neck is useful in assessing mucosal swelling of the oral cavity, mucosal pharyngeal space, larynx, and extra-mucosal spaces of the subcutaneous soft tissue specifically affected in ACE-i angioedema. Airway compromise can also occur, which may require emergent intubation. Treatment involves airway management and cessation of the medication, and angioedema subsequently resolves in 48-72 hours.
